# How to achieve sustainable buyer–seller relationship in social commerce? The effect of network closure on ties evolution

**DOI:** 10.3389/fpsyg.2022.1104770

**Published:** 2023-01-13

**Authors:** Hao Zhang, Xiao Han, Shiyong Zheng, Mohan Gu

**Affiliations:** ^1^School of Economics and Management, Hubei University of Technology, Wuhan, China; ^2^Hubei Circular Economy Development Research Center, Wuhan, China; ^3^School of Management, Wuhan University of Technology, Wuhan, China; ^4^School of Business, Guilin University of Electronic Technology, Guilin, Guangxi, China; ^5^College of Economics and Management, Huazhong Agricultural University, Wuhan, China

**Keywords:** social commerce community, network closure, social influence, social selection, social ties

## Abstract

The fact that most buyer–seller ties in the social commerce community are easy to form but hard to keep has brought the “social bubble” into social commerce. Following the literature streams of network closure and social commerce and based on the longitudinal dataset of an online social commerce community over a year, this article explores the buyer–seller ties evolution in the social commerce community through two stages, that is, ties emergence versus ties persistence. In this study, the authors build a hazard model and estimate with a semiparametric partial likelihood method. Our results show an asymmetric effect of network closure mechanisms across different stages of buyer–seller ties evolution. In the early stage of buyer–seller ties, due to the information asymmetry, buyers usually rely on informative signals that either reflect the “popular others” (i.e., the popularity and content sharing) or the “ideal self” (i.e., the value homophily and status homophily) to form ties with sellers, which makes the community more “transactional.” As very few ties can survive through the periods of 3 months or more, the normative social influence, which relies heavily on the structure of extant relationships among community members, becomes the dominant driver of ties persistence, which makes the community more “social.” This study contributes to the ongoing research of network analysis and social commerce. It provides valuable tactics to sellers who want to develop long-term relationships with buyers in the social commerce community.

## Introduction

Social commerce communities have recently emerged as important online shopping channels because they have a unique advantage in bridging the gap between transactions and social interactions among community members as potential buyers and sellers. According to recent statistics in eMarketer (2022), consumers spend $15.1 billion *via* social commerce channels in the United Kingdom in 2022. That’s 5.5 million more than in 2019, before the pandemic. Sellers participate in social commerce communities for commercial and social purposes (Xiao et al., 2015; Su et al., 2021). With the belief that more followers can trigger a “network effect” on their market performance, e.g., network externality (the ability to attract more buyers from outside the network; Cao and Li, 2020) and social contagion (the ability to increase the cohesion or adoptions from within the network; Harmeling et al., 2017; Yokotani and Takano, 2021).

While both scholars and market practitioners have acknowledged the tremendous economic and social value of expanding social commerce communities, a few critical yet unsolved issues have emerged and hindered the development of the social commerce community. For example, according to our dataset, a surprisingly high rate of ties emergence is found. That is, 3,461 of the total buyers in our sample (82%) had formed 55,376 ties with 1,007 sellers throughout the observation. Among which 66% of the buyer–seller ties had been “broken”^1^ within only 1 week, 21% can survive through one to 3 months, leaving only 13% can persist as long as more than 3 months. This mere “flash in the pan” phenomenon has brought the “social bubble” into social commerce and misled relationship management in social commerce communities. Although past studies have suggested that the number of online followers alone can exert a network externality effect on the market, it is still essential to understand what drives the long-term relationship of each buyer–seller tie in the context of the social commerce community. For example, Ryals and Payne (2001) find that when the customer retention rate increases by 5%, the company’s profitability (i.e., net present value NPV) increases from 20 to 85%. Xiao et al. (2015) argue that sellers can improve visibility and sales performance by building buyer relationships. Homburg et al. (2017) suggest that stable customer relationship is the key to creating long-term value for enterprises. Therefore, sellers need to trade off between the market efforts in attracting new followers and retaining their existing followers to maximize their market performance. Due to the dynamic nature of online relationships, the mechanisms that drive the emergence and persistence of social ties in the social commerce community should be varied. Extant research has employed the network closure theory to account for online relationship emergence from a static perspective (Steinhoff et al., 2019; Smith and Smith, 2021). However, whether the critical factors in network closure (i.e., the external and internal drivers) play the same role in the evolution of buyer–seller ties in the social commerce community is still questionable. This is important because the fundamental nature of the social commerce community may dramatically change (e.g., from being more transactional to being more social) as the buyer–seller ties evolve, and the sellers may adjust their relationship strategies toward the potential buyers.

To address these gaps in the literature and practice, this research explores the evolution mechanism of social business communities in different stages of relationship interaction from the perspective of dynamic evolution. Our findings suggest that the influence of network closure mechanisms is asymmetric to varying stages of the evolution of buyer–seller relationships. Specifically, internal factors such as value homogeneity and status homogeneity play the most important role in the emergence of ties. In contrast, external factors, primarily normative social influences, are the key drivers that sustain buyer–seller relationships over a more extended period, especially in the later stages of development. Therefore, the best way to keep ties with buyers in the social commerce community is to build a relationship with their followers or followees so that the group norm will motivate them to maintain the already existing buyer–seller relations. Therefore, the best way for sellers in social business communities to stay connected with buyers over time is to connect with the buyers’ followers or followees, and group norms will drive buyers to visit and connect with sellers.

The rest of the paper is set out as follows. Section 2 reviews the network closure theory and introduces the hypotheses. In Section 3, we explain our data and method. Our data included an online social commerce community that contains the online behaviors of 4,221 buyers and 1,210 sellers over a year. We build a hazard model and estimate with a semiparametric partial likelihood method based on the datasets. In Section 4, we test our main hypotheses and give conclusions and discussions in Section 5.

## Theoretical background and hypotheses

### Revisit of network closure theory

In its early works, network closure describes the underlying mechanism that drives social ties to form and evolve from a minimal social entity, e.g., the dyadic closure or the triadic closure (e.g., Burt, 1987, 2004; Coleman, 1990). They regarded network closure as capital because it does bring two things to people in the closed networks. First, people acquire a wealth of information from others in their social relationships, and a basic form of social capital is the information potential in social relationships. Second, it will reduce the risk of trusting others in interpersonal communication to facilitate the effective conclusion of transactions or decisions.

In sociological research, scholars have richly explored the influence mechanisms of network closures. Coleman (1990) stated that network closure stresses the role of cohesive ties in fostering a normative environment that facilitates cooperation. The normative influence in the social media context refers to the established social ties driven by emotional drivers (Kent and Li, 2020). As more scholars realize the role that informative ties play in the social context, Aral (2016) and Aral and Dhillon (2022) viewed cohesive ties as rigidity. They focused on the “weak tie” that can bring novel information outside the cohesive and normative social group.

Along with the increasing computational capacities, interactions among an entire or sampled community members are usually visualized. Varied patterns and effects of network closure are identified from both macro (global features of the network; Allcott et al., 2007) and micro (ego-network features; Kossinets and Watts, 2006; Van den Bos et al., 2018) perspectives. The network closure theory is applied in interpersonal relations analysis and provides in-depth mechanisms to explain inter-organizational relations. For example, a recent study by Amati et al. (2021) discusses inter-organizational network closure mechanisms from the perspective of multilevel (hierarchical) relationships.

Due to the heterogeneity of ties among network members, the existing literature suggests that network closure can be driven either externally or internally (Kossinets and Watts, 2006; Xiao et al., 2015). The research stream focuses on external influence (e.g., reciprocity, contagion, observational learning, etc.). It considers that people in social relationships are primarily influenced by informational or normative influences, which lead to the alignment of ideas or behaviors within the community (Kossinets and Watts, 2006; Park et al., 2018). The research stream focuses on the external influence (e.g., structural equivalence and homophily, etc.) and emphasizes the selection effect of community members, with the general perception that members befriend those who share similar characteristics with them (Dev, 2016; Peixoto, 2022). Scholars have noted that relationships evolve dynamically, but previous studies have not distinguished between the different effects of different drivers at different stages of the relationship.

Above all, we illustrate the literature on the network closure theory and highlight our focus and contributions in Table 1.

**Table 1:** Selective literature review of network closure theory.

Literature	Research question	Research subjects	Main variables	Research context	Research perspectives	Research method
Coleman (1990)	Persons’s subjective approach to institutions	Social institutions	Institutinal structure; common value; private interests	Offline	Static	Commentary
Kossinets and Watts (2006)	Social networks evolve	Students faculty staff	Social network; social ties	Offline	Dynamic	Empirical analysis
Allcott et al. (2007)	Prosocial behaviors	Community	Community size; network structure	Offline	Static	Modeling
Xiao et al. (2015)	Relationship formation	Social commerce community	Social network; network closure	Online	Static	Empirical analysis
Aral (2016)	Novel information	information	Week ties	Offline	Static	Theoretical overview
Dev (2016)	Community structure underlying a net-work	Village	Homophilous; group; community; structure	Offline	Static	Empirical analysis
Park et al. (2018)	Users’ spending behavior	Online role-playing game community	Social dollars; social contagion; network density	Offline	Static	Empirical analysis
Van den Bos et al. (2018)	Prosocial behavior	School class	Social cohesion; social behavior	Offline	Static	Online survey
Amati et al. (2021)	Co-evolution of organizational and network structure	Health care organizations	Network evolution; organizational change	Offline	Dynamic	Modeling
Aral and Dhillon (2022)	Information flow	Email network	Network; knowledge transfer	Online	Dynamic	Modeling + empirical analysis
Peixoto (2022)	Network homophily	Community	Community structure; triadic closure	Online	Static	Modeling
This paper	Buyers-sellers relationship	Social commerce community	Social network; popularity; content sharing	Online	Dynamic	Modeling + empirical analysis

### External drivers in network closure

The external factors mainly come from an informative or normative social influence (Wang et al., 2019). The informative social influence works when users of social network sites want to receive novel and up-to-date information from their followers (Chen et al., 2016). In work by Burt (2004) and Coleman (1990), they point out that the members of a group, who are completely unfamiliar with each other, will refer to visible signals to accumulate knowledge about the foreign environment. And acquire the necessary information and knowledge in environmental cognition through interpersonal interactions (e.g., invitation, communication, etc.). Therefore, under the informational social influence, the evolution of network relationships often results in the concentration of relational resources, that is, the scale-free property of network structure (Lee et al., 2011).

Furthermore, in a social commerce community consisting of strangers, a member with signals of expertise or popularity (e.g., having more followers or more content sharing) is perceived as informative and thus preferred and followed. As another external driver of network closure, the normative social influence can enhance the cohesiveness among a group of community members. When the members are embedded into a local network (such as organizations, communities, specific regions, etc.), their behaviors are influenced by the norms of their embedded network. That is, through identifying group values to achieve the purpose of self-preservation and reinforcement, or through behavioral compliance to obtain rewards for the group. Therefore, under normative social influence, social relations usually evolve toward clustering and conformity (Lee et al., 2011; Cho and Chan, 2021).

### Internal drivers in network closure

The internal factors that drive network closure are rooted in the literature on selection effects (Simon, 1990). In their work regarding behavioral dynamics, Steglich et al. (2010) raise the recurrent problem of separating the effect of selection from the social influence in partner selection. They suggested that selection effects increase the probability of interacting with people with the same characteristics. This phenomenon of people selecting their social ties homophilous was formally introduced by Kossinets and Watts (2009). Therefore, the internal driver of homophily, which stresses the “birds of a feather flock together, “is usually confounded with the external driver of social influence, which emphasizes the contagious process of a focal member starting to have specific characteristics they do not have in the first place. Extensive literature has since emerged on the internal drivers in social relations. For instance, Centola and van de Rijt (2015) conducted a more in-depth discussion on the social selection mechanism and proposed two types of social selection mechanisms. First, people may construct some enterprising ties because of the homogeneity of values. For example, members in the network will set goals (such as fitness goals, skill training goals, etc.) according to their interests and hobbies and choose other members that are consistent with their future goals to construct relationships. Second, members in the network may also emerge in similar attribute relationships because of state homogeneity. For example, members in the network will select members with certain similarities to build relationships based on their gender, age, social class, and other attributes (McPherson et al., 2001). In a specific empirical research, Centola and van de Rijt (2015) discussed how members of an online health organization (an informal social organization where members are unfamiliar with each other) construct relationships based on their attributes (status homophily; such as gender, age, geographic location, etc.) and health goals (value homophily; fitness plan, health problems to overcome, etc.).

### Network closure from a dynamic perspective

It is worth noting that the above literature on network closure usually applies a static view on network closure since they are preoccupied with the emergence of ties. At the same time, the emergence of ties has enormous managerial implications, especially in social marketing, where market revenues come directly or indirectly from the number of online buyer–seller ties. It is still worth the effort to look further beyond ties emergence in that the stability of online buyer–seller ties is the key to creating long-term value for enterprises (Gupta et al., 2019). Among the few studies on the dynamics of network closure, Kossinets and Watts (2006) studied the interpersonal network closure among newly enrolled students in a university through a semester-long observation. However, no further discussion is provided on whether or how the network closure among the students would persist for a specified period. Dahlander and McFarland (2013) studied the emergence and persistence of interpersonal network closure in the organizational context. They found that in the emergence stage of the network closure, members in the same organization chose to build cooperative ties based on popularity and tie strength with their targets. Therefore, we hypothesize that:

*H1*: The network closure in the tie emergence stage can be driven externally, that is, the signals of (a) popularity (e.g., number of followers) and (b) content sharing (e.g., number of posts and replies) will positively increase the probability of network closure from buyers to sellers.

While in the long run, the common organizational goals and cooperation experience determined whether these cooperative ties would persist. Nevertheless, in a social commerce community where the members share no explicit organizational goals, whether and how the drivers of network closure in the emergence stage still work in the ties persistence stage remains unknown. When buyers in the social commerce community start to build ties with others, information asymmetry makes it difficult for them to identify suitable partners. Buyers as new members in a social commerce community usually follow the trend and build ties with the sellers who already have more followers (known as a preferential attachment; Zhang et al., 2018) or more posts on the community forum to show their expertise and increased impressions. Buyers also choose their targets according to the degree of match between the signals they observe from the sellers and their characteristics, known as an internal homophilous process in network closure (Xiao et al., 2015). As the members engage in increased community activities, the buyers’ increased embeddedness makes the online community evolve from primarily transactional to more relational (Walker and Lynn, 2013; Zhang et al., 2018). Therefore, we hypothesize that:

*H2*: The network closure in the tie emergence stage can also be driven internally, that is, the signals of (a) value homophily (e.g., the content of posts and replies) and (b) status homophily (e.g., the information displayed on sellers' homepage) will positively increase the probability of network closure from buyers to sellers.

Meanwhile, the social norm develops within the local networks of the buyers, which may dramatically change the drivers and mechanisms of the network closure. According to Burt (2004), the social norm usually works in the social network through cohesion or structural equivalence. The cohesion approach refers to the socialization between the ego (tie sender) and alter (tie receiver) based on their direct and empathic communications. In contrast, the structural equivalence highlights the similarity of social roles between ego and alter based on their structural properties in the network. Specifically, in our social commerce community context, under the cohesion approach, the buyers in the social commerce community usually keep their ties with whom their online friends (i.e., followers) have kept ties because their friends act as relationship references. Their social relations can be perceived as the implicit social norm within the local networks of the buyers (Kozlenkova et al., 2017). While under the structural equivalence approach, the buyers in the social commerce community usually keep their ties with whom they share with similar relationship structure because the similar relationship (i.e., shared followers or followers) usually indicates similar tastes, identity, or possible cooperation in the future (Kamis et al., 2018), which also acts as the social norm to make the buyers keep their ties with their structurally equivalent sellers. Therefore, we hypothesize that:

*H3*: The network closure in the ties persistence stage is driven mainly by the normative social influence. Specifically, we expect positive effects of (a) relational reference and (b) structural equivalence on the buyer-seller ties persistence.

## Data and method

### Data collection and pre-processing

Our data come from one of the biggest E-commerce platforms in China, Taobao.com. A web crawler was programmed using Python 3 to search and store data from the chosen online community. Since the website does not disclose the exact time of ties’ emergence (or disappearance), we upload our web crawler program to a server to automatically track the ties dynamics among the community members on a daily base. By doing this, we can get snapshots of the ties’ evolution among the community members and get the tie emergence and duration by comparing adjacent relationship snapshots.

### Measurements

The ties emergence is a binary variable that equals 1 if a tie is formed between a buyer *i* and a seller *j* at the time *t* and 0; otherwise, the ties persistence is a series of binary variables on different conditions. Specifically, for *D* = 1, $TP_{i,j,t,D}$ equals to 1 if the duration of the tie between member *i* and *j* lies between 1 week and 1 month (a representative for short persisted ties) and 0 otherwise; for *D* = 2, $TP_{i,j,t,D}$ equals 1 if the duration lies between 1 month and 3 months (a representative for medium persisted ties) and 0 otherwise; for *D* = 3, $TP_{i,j,t,D}$ equals 1 if the duration exceeds 3 months (a representative for long persisted ties) and 0 otherwise. Another important issue in measuring the ties’ persistence is the criteria for ties ending. Most marketing managers define the end of a buyer–seller tie as buyers actively delinking from previously formed ties. In practice, many buyers “end” a pre-existed tie without doing anything. While we still take the active delinking as a measure of ties ending, we also include another measurement: for those who had already stopped interactions but never delinked with their followers or followings. We take their last interactions (i.e., the last reply or “@” in the community forum) as the dates of ties ending.

There are two types of informational signals in the social commerce community that the community members can observe: (a) Popularity (PO), which is a common measurement in most social network studies, is measured as the in-degree, that is, the number of followers a community member has already attracted in the platform; (b) Content sharing (KS), which is also a ubiquitous measurement to reflect the member’s capability and expertise in a social commerce community, is measured as the number of posts and replies in our research context. The normative social influence focuses on the unobservable relationship structure among the community members. Specifically, relevant research has identified and measured two types of normative social influence based on community members’ embedded social structure: (a) Relational reference (RR), which refers to the extent to which a community member would imitate his or her “friends” (followers or followees). (b) Structural equivalence (SE) has been studied in most social network literature since the seminal work of Burt (1987) and Coleman (1990). It is measured as the common friends between buyer i and seller j before they form or persist a tie in our research.

According to previous research regarding the definition of social selection (Simon, 1990; Steglich et al., 2010), We measure the internal effect of social selection from two major approaches: (a)Value homophily (VH) is measured as the number of joint participation between buyer *i* and seller *j* (e.g., posts and replies) under the certain topic in the community forum. (b) Status homophily (SH) is measured as the Euclidean distance between *i* and seller *j* on many demographic properties.

### Model setup and specifications

In general, ties’ emergence or persistence is a time-based binary event, and the probability of ties’ evolution over time is a function of a series of time-varying covariates. It is reasonably argued that standard regression approaches are unsuitable for analyzing such survival times because such data are typically right-censored (i.e., not all community members form a tie with everyone else by the end of the observation period). While such time-based phenomena are usually modeled effectively with a hazard function, which can investigate the effects of the covariates both cross-sectionally and longitudinally, as well as handle the sample selection bias that may be caused by the censoring data (Mitra and Golder, 2002). In our study, we build a hazard model and estimate with a semiparametric partial likelihood method (Thompson and Sinha, 2008). Note that unobserved heterogeneity has a potential role in our analysis, and the most popular technique to control for unobserved heterogeneity is the random-effects specification on the error term (Vaupel et al., 1979). Specifically, the basic hazard model is set up as follows:


(1) $$h\left(t|x_{i,j,t_{ij}}\right)=\underset{\Delta t\to 0}{\lim}\frac{Pr{\left(t_{ij}\leq T\leq t_{ij}+\Delta t|T\geq t_{ij}\right)}_{ij}}{\Delta t}$$

where $\mathrm{Pr}{\left(t\right)}_{ij}$ is the accumulated distribution function for the buyer *i* following the seller *j*. It is affected by the following covariates:


(2) $$h\left(t_{ij}|x_{i,j,t_{ij}}\right)=h_{0}\left(t\right)\exp\left(x_{i,j,t}\beta \right)$$

where $h\left(t|x_{i,j,t}\right)$ is the hazard of tie emergence between *i* and *j*. It represents the instantaneous probability of tie emergence between *i* and *j* given that it has not occurred yet at the time t; $h_{0}\left(t\right)$ is the baseline of tie emergence hazard; $x_{i,j,t}$ is a row vector of covariates that indicate *i* follows *j* at the time *t*, which mainly consists of the antecedents (and their interactions) we argued previously. Besides, unobserved heterogeneity can also affect the tie emergence hazard between *i* and *j*. For example, some buyers are intrinsically more or less likely than others to build ties with the seller. Therefore, we follow the method of Winship and Mare (1992) and specify the random effect that follows a particular functional form of unobserved characteristics in the hazard model as follows:


(3) $$h\left(t_{ij}|x_{i,j,t_{ij}}\right)=h_{0}\left(t\right)\exp\left(x_{i,j,t}\beta +\tau_{ij}\right)=h_{0}\left(t\right)\exp\left(x_{i,j,t}\beta \right)\Psi \left(\theta \right)$$

where $\Psi \left(\theta \right)=\exp\left(\tau_{ij}\right)$ represents the unobserved heterogeneity that operates in a multiplicative manner on the basic hazard model. Thus, the full hazard model with all the covariates specified can be given:


(4) $$h\left(t_{ij}|x_{i,j,t_{ij}}\right)=h_{0}\left(t_{ij}\right)\exp\left(\begin{array}{l} \beta_{m1}PO_{i,j,t}+\beta_{m2}CS_{i,j,t}+\beta_{m3}RR_{i,j,t}\\ +\beta_{m4}SE_{i,j,t}+\gamma_{m1}VH_{i,j,t}+\gamma_{m2}SH_{i,j,t}\\ +\mu_{m}\sum Interactions+\delta_{m}\sum Controls+t_{ij} \end{array}\right)$$

where $PO_{i,j,t}$ , $KS_{i,j,t}$ , $RR_{i,j,t}$ , and $SE_{i,j,t}$ represent the popularity, content sharing (informative social influence), relational reference, and structural equivalence, respectively, and $\beta_{m1-4}$ are their associated coefficients. $VH_{i,j,t}$ and $SH_{i,j,t}$ represent the value homophily and status homophily, respectively, and $\gamma_{m1-2}$ are their associated coefficients. Since the experience of community members usually plays an important role in their community behaviors, we also include this factor as interactions with the main covariates to account for the difference of the tie emergence and persistence for members with varied community experience and $\mu_{m}$ is a vector of the associated coefficients. Finally, $\delta_{m}$ is a vector of the associated coefficients for control variables; note that *m* is a model indicator, *m* = 1 represents the ties emergence hazard model, and m = 2,3,4 represent the ties persistence hazard model where the duration of ties lies between 1 week and 1 month, 1 month and 3 months, and more than 3 months, respectively.

## Model estimation and results

### Descriptive statistics

As we explore the tie emergence vs. ties persistence in an online social commerce community, several datasets are compiled from the raw data we collected from May 1, 2016 through May 1, 2017. We list the sample size of each dataset as well as their network characteristics in Table 2.

**Table 2:** Descriptives of datasets in ties formation versus persistence analysis.

Datasets descriptions	Dataset 1 (ties formation)	Dataset 2 (less than a week)	Dataset 3 (1 week, 3 months)	Dataset 4 (more than 3 months)
Sample size	5,431 (4,221 buyers 1,210 sellers)	4,468 (3,461 buyers 1,007 sellers)	2,201 (1,629 buyers 572 sellers)	922 (704 buyers 218 sellers)
Formed/broken Buyer–seller ties	55,376	36,548	11,627	7,201
Mean indegree	11.67	13.24	14.23	13.56
Mean outdegree	14.89	16.75	15.84	15.17
Mean posts	13.45	15.34	17.55	16.34
Mean replies	16.78	17.25	18.69	17.61
Total of ties	114,352	111,356	17,356	14,521
Network diameter	12	11	8	6

According to the descriptive statistics in Table 1, fewer community members enter the dataset as the buyer–seller ties evolve from emergence to persistence. The acceleration of ties dissolving is surprising: among all the ties formed in our sample (3,461 buyers formed 55,376 ties with 1,007 sellers), only 704 buyers maintained their ties with 218 sellers after 3 months of the ties emergence. This contrast of a high rate of emergence but a low rate of persistence reveals both the importance and hardship of relationship management in social commerce communities. Unlike the typical social media of Facebook, people intend to maintain longer relationships based on a series of factors, such as offline interactions and embedded social roles of friends, families, or colleagues. The social commerce community consists of buyers and sellers who intend to build ties to reduce information asymmetry or seek peer recommendations. The commercial nature and online anonymity of the social commerce community make the ties among its community members form and end quickly. In the following part, we dig into the mechanisms that drive tie dynamics in the social commerce community through our models.

### Ties emergence model estimation

We begin with the basic proportional hazard model (PH model) estimation using the partial likelihood procedure (Rubio et al., 2021) in R 3.6.5, the estimation package used here is “survival.” Next, we estimate the equation (4) to control for unobserved heterogeneity. The results of the model estimation procedure are reported in Table 3.

**Table 3:** Ties emergence model estimation and results.

IVs	Cox PH Model (baseline hazard model)		Weibull hazard model (with unobserved heterogeneity)	
	Main effect only	Full model	Main effect only	Full model
**Informative social influence**				
Popularity (PO)	0.135 (0.006)**	0.122 (0.011)**	0.183 (0.009)**	0.212 (0.006)**
Content sharing (CS)	0.129 (0.011)**	0.113 (0.008)**	0.164 (0.012)**	0.077 (0.093)
**Normative social influence**				
Relationship reference (RR)	0.073 (0.151)	0.031 (0.167)	0.007 (0.143)	0.016 (0.101)
Structural equivalence (SE)	0.006 (0.016)	0.023 (0.063)	0.001 (0.057)	0.004 (0.067)
**Social selection**				
Value homophily (VH)	0.025 (0.003)**	.351(.032)**	0.086 (0.016)**	0.271 (0.013)**
Status homophily (SH)	0.049 (0.029)	0.243 (0.014)**	0.172 (.007)**	0.007 (0.033)
**Interactions**				
Buyer experience × PO	-	−0.053 (0.008)**		−0.146 (0.068)**
Buyer experience × KS	-	−0.127 (0.005)**		−0.427 (0.021)**
Buyer experience × VH	-	0.117 (0.007)**		0.125 (0.018)**
Buyer experience × SH	-	0.013 (0.001)**		0.036 (0.004)**
**Controls**				
Buyer experience	0.012 (0.006)**	0.016 (0.008)**	0.024 (0.129)	0.126 (0.172)
Buyer’s previous followers	0.018 (0.162)	0.008 (0.063)	0.031 (0.244)	0.016 (0.379)
Buyer’s previous followees	0.248 (0.003)**	0.312 (0.041)**	0.301 (0.038)**	0.253 (0.017)**
Seller’s rating	0.419 (0.017)**	0.309 (0.034)**	0.425 (0.044)**	0.262 (0.012)**
Shape parameter *p*	-	-	3.154 (0.564)	3.563 (0.327)
Test of H_0_: θ = 0	-	-	*p* < 0.01	*p* < 0.01
**Model evaluations**				
Adjusted *R*^2^	0.23	0.25	0.29	0.31
Log-likelihood	−16021.741	−15747.127	−15087.563	−14185.419
AIC	30087.957	29769.018	27039.021	24106.837
BIC	30138.713	29769.007	27856.846	24296.044

*p < 0.05, **p < 0.01.

Table 3 reports the results for the drivers of ties emergence in the social commerce community. The results across the Cox PH model and the Weibull hazard model with unobserved heterogeneity are consistent, and all the specification tests suggest the importance of controlling for unobserved heterogeneity in the ties emergence dataset (the test of the null hypothesis that θ = 0 is rejected in each Weibull hazard model). Thus, the following discussion on ties emergence relies on the results of the Weibull hazard model. Notably, in each Weibull hazard model, the shape parameter *p* is always greater than 1, implying that a monotonically increasing hazard exists during our observation period. This is in accordance with the basic assumption in applying the hazard model that the probability of a dyadic tie between buyer and seller should monotonically increase (or decrease) as time goes by. Only the Weibull hazard model accounts for 29% of the variance in ties emergence after controlling the observed and unobserved heterogeneities. Specifically, in support of *H*1 and *H*2, the informative social influence and social selection factors are the main drivers, and the normative social influence is insignificant in the ties emergence stage ( $\beta_{11}$ =0.183, p<0.01 ; $\beta_{12}$ =0.164, p<0.01 ; $\gamma_{11}$ =0.086, p<0.01 ; $\gamma_{12}$ =0.172, p<0.01 ). These results provide support for the conceptual argument that in the early age of ties evolution in the social commerce community, due to the information asymmetry, members mostly rely on informative signals that either reflects the “popular others” (i.e., the popularity and content sharing) or the “ideal self” (i.e., the value homophily and status homophily) to form ties with others. Besides, buyers’ experience is also important in this ties emergence stage. Specifically, after we control for the buyers’ experience and its interactions with the social influence and social selection factors, the model gets better model evaluation statistics (adjusted *R*^2^ increases from 29 to 31%, and the AIC and BIC decrease accordingly). As the buyers gain more experience in the social commerce community, the effects of the informative signals of popularity and content sharing significantly decrease ( $\mu_{11}$ = − 0.146, p<0.01 ; $\mu_{12}$ = − 0.427, p<0.01 ) while the effect of the informative signals of value homophily and status homophily significantly increase ( $\mu_{13}$ =0.125, p<0.01 ; $\mu_{14}$ =036, p<0.01 ). We can further infer from these interaction effects that experienced buyers in the social commerce community rely more on the signals that better express themselves even in an early stage of their ties with others (i.e., ties emergence stage). Therefore, the sellers must engage with buyers differently based on their varied community experience, even in the early stage of the seller–buyer ties.

### Ties persistence model estimation

The model estimation procedure for ties persistence is similar to the ties emergence. The only difference is that the dependent variable, the hazard event of ties persistence, varies according to the period of ties persistence. We get the results of different periods of tie persistence by running the associated model on each dataset we introduced in Table 4.

**Table 4:** Ties persistence model estimation and results.

Independent Variables	Cox PH Model (baseline hazard model)			Weibull hazard model (with unobserved heterogeneity)		
	Model 1A (<1 week)	Model 2A (1 week, 3 months)	Model 3A (>3 months)	Model 1B (<1 week)	Model 2B (1 week, 3 months)	Model 3B (>3 months)
**Informative social influence**						
Popularity	0.033 (0.011)**	0.027 (0.049)	0.019 (0.063)	0.021 (0.032)	0.016 (0.027)	0.005 (0.012)
Content Sharing	0.047 (0.072)	0.034 (0.014)**	0.007 (0.018)	0.031 (0.007)**	0.021 (0.011)	0.014 (0.039)
**Normative social influence**						
Relationship reference	0.014 (0.012)	0.023 (0.029)	0.028 (0.002)**	0.007 (0.019)	0.114 (0.008)**	0.576 (0.012)**
Structural equivalence	0.009 (0.015)	0.015 (0.003)**	0.019 (0.004)**	0.003 (0.024)	0.216 (0.013)**	0.733 (0.003)**
**Social selection**						
Value homophily	0.016 (0.031)	0.022 (0.006)**	0.017 (0.023)	0.036 (0.012)**	0.025 (0.037)	0.012 (0.017)
Status homophily	0.021 (0.014)	0.011 (0.018)	0.007 (0.014)	0.176 (0.014)**	0.028 (0.059)	0.016 (0.063)
**Interactions**						
Buyer experience x RR	0.036 (0.052)	0.104 (0.093)	0.056 (0.002)**	0.062 (0.014)**	0.159 (0.014)**	0.079 (0.013)**
Buyer experience x SE	0.162 (0.025)**	0.167 (0.087)	0.203 (0.005)**	0.178 (0.012)**	0.394 (0.019)**	0.241 (0.004)**
**Controls**						
Buyer experience	0.034 (0.043)	0.049 (0.006)**	0.186 (0.026)**	0.046 (0.008)**	0.103(0.027)**	0.235 (0.038)**
Buyer’s previous followers	0.012 (0.002)**	0.015 (0.019)	0.024 (0.009)**	0.008 (0.013)	0.013(0.005)**	0.016 (0.003)**
Buyer’s previous followees	0.101 (0.213)	0.176 (0.034)**	0.078 (0.005)**	0.065 (0.002)**	0.124 (0.018)**	0.132(0.037)**
Seller’s rating	0.086 (0.007)**	0.015 (0.068)	0.042 (0.035)	0.057 (0.011)**	0.019 (0.031)	0.007 (0.012)
Shape parameter *p*	-	-	-	7.144 (0.124)	7.467 (0.187)	7.841 (0.092)
Test of H_0_: θ = 0	-	-	-	*p* < 0.01	*p* < 0.01	*p* < 0.01
**Model evaluations**						
Adjusted *R*^2^	0.21	0.23	0.25	0.22	0.26	0.33
Log-likelihood	−15987.563	−14521.952	−13876.822	−15125.331	−13158.431	−10547.884
AIC	29939.026	28501.347	27862.982	29035.655	27022.621	24564.049
BIC	27984.857	25257.334	24775.109	27155.611	24118.756	21772.423

*p < 0.05, **p < 0.01.

In Table 4, we present the results of ties persistence analysis based on the nested models. From model 1 to model 3, we focus on varied lengths of periods of ties persistence in the social commerce community. Specifically, model 1 focuses on the relatively short persistence of ties between buyers and sellers (i.e., the ties that last for less than a week); model 2 focuses on the median persistence of ties that last for more than a week and less than 3 months; model 3 focused on the long persistence of ties that last for more than 3 months. From model “A” s to model “B” s, we compare their results to see whether more complicated models with specifications of unobserved heterogeneity outperform the benchmark models. The specifications tests suggest the importance of controlling for unobserved heterogeneity in all ties persistence datasets (The tests of the null hypothesis that θ = 0 are rejected in model 1B, 2B, and 3B). Thus, the following discussions on ties persistence will rely on the results of the Weibull hazard models.

As the social commerce community buyers intend to maintain more extended ties with sellers, the significance and effect size of social influence and social selection change dramatically. Specifically, in support of *H*3, the effect size and significance of normative social influence are increasing throughout the ties persistence stage ( $\beta_{23}$ =0.007, *p* > 0.05, $\;\beta_{33}$ =0.114, *p* < 0.01, $\beta_{43}$ =0.576; *p* < 0.01; $\beta_{24}$ =0.003, *p* > 0.05, $\;\beta_{34}$ =0.216, *p* < 0.01, $\beta_{44}$ =0.733, *p* < 0.01) while the effect of informative social influence diminishes ( $\beta_{21}$ =0.021, *p* > 0.05, $\;\beta_{31}$ =0.016, *p* > 0.05, $\beta_{41}$ =0.005, *p* > 0.05; $\beta_{22}$ =0.031, *p* < 0.01, $\;\beta_{32}$ =0.021, *p* > 0.05, $\beta_{42}$ =0.014, *p* > 0.05). Besides, the effects of social selection factors also diminish ( $\beta_{25}$ =0.036, *p* < 0.01, $\;\beta_{35}$ =0.025, *p* > 0.05, $\beta_{45}$ =0.012, *p* > 0.05; $\;\beta_{26}$ =0.176, *p* < 0.01, $\;\beta_{36}$ =0.028, *p* > 0.05, $\beta_{46}$ =0.016, *p* > 0.05). Note that although fewer variables hold significance in the long period ties persistence hazard model, the overall model evaluation is remarkably better (Adjusted *R*^2^ = 0.33, and both the AIC and BIC in Model 3B outperform the other models), which indicates a simple long-term relationship management principle in social commerce community: build social norm through possible relationship strategies. Therefore, sellers should establish ties with not just certain buyers but their followees (the effect of relational reference) or followers (the effect of structural equivalence) in order to increase the social norm within the embedded community members. Besides, the control variable of buyers’ experience always plays a significant and positive role throughout the ties’ persistence periods. As buyers gain experience, the positive effects of normative social influence on their ties persistence will be enhanced ( $\mu_{21}$ =0.062, p<0.01 ; $\;\mu_{31}$ =0.159, p<0.01 ; $\;\mu_{41}$ =0.079, p<0.01 ; $\;\mu_{22}$ =0.178, p<0.01 ; $\;\mu_{32}$ =0.394, p<0.01 ; $\;\mu_{42}$ =0.241, p<0.01 ). This finding provides insight into the relationship management for experienced buyers in the social commerce community. That is, as the buyers stay longer in a social commerce community, they intend to maintain stable ties with sellers under the effect of normative social influence.

## Discussion

### Findings and contributions

As a relatively new phenomenon, social commerce has evolved quickly in practice and academic research (Xiao et al., 2015; Ko, 2020; Liao et al., 2022). The social commerce community is a marriage of transactional and social that enables its members to get their fans and followers shopping by making the whole selling process social. However, we know little about how the social commerce community evolves from transactional to social. There is uncertainty and instability regarding the buyer–seller ties through this process. Extant research has focused on forming the ties between buyer and seller in the social commerce community. Still, less is known about how the ties evolves through different stages. We advance this research stream by applying the network closure theory from a dynamic view and digging into each stage of the tie development and its mechanism. A web crawler is programmed using Python 3 to track the online behaviors of members in an online social commerce community from May 1, 2016, to May 1, 2017. Subsequently, we compile several datasets from the same social commerce community based on the varied durations of ties. We run the hazard models to test the network closure between buyers and sellers in different stages of ties evolution. Several theoretical contributions and managerial implications are discussed based on the results of our analyses.

This study contributes to social network analysis studies in social shopping and social commerce communities. While the social network has been well established in the context of offline community, an emerging stream of research in marketing focuses on the evolution of social ties in the online community (e.g., Xiao et al., 2015; Dev, 2016; Park et al., 2018; Aral and Dhillon, 2022, etc.). Our study contributes to the limited research that examines the evolution of the social ties between the buyers and the sellers at different relationship stages in the online social commerce community. Generally, our results show that the network closure theory can be applied to account for ties development between buyers and sellers in the social commerce community. Still, not all the factors are significant throughout the whole lifetime of buyer–sellers ties. Given the instabilities of buyer–seller ties in the social commerce community (Stanko et al., 2007; Gupta et al., 2019), it is necessary to revisit the network closure theory with a dynamic view to explore the varied mechanisms that drive ties emergence versus persistence. We can conclude from our results that although the effects of network closure factors hold across different stages of ties evolution, their significances and effect sizes change dramatically. Specifically, internal factors such as value homophily and status homophily play the most critical roles in the emergence of ties.

In contrast, external factors, primarily normative social influence, are critical drivers to maintaining buyer–seller ties for longer. Despite the transactional nature of the online social commerce community, the social factor of normative influence is surprisingly significant and positive in the later stage of buyer–seller ties development. These investigations of the dynamics of buyer–seller ties extend previous research and prove that the network closure theory can not only be applied to predict ties emergence (Xiao et al., 2015; Gupta et al., 2019) but also the long-term relationships between buyers and sellers in social commerce communities. However, it is necessary to realize the temporal changes in the network closure process’s significance and effect size of internal versus external factors.

Our findings also provide important managerial implications for market practitioners who want to develop long-term relationships with buyers in the social commerce community. The ties in social commerce are very different from those in social media platforms such as Facebook and Twitter because buyers in social commerce are usually more goal-directed and motivated to buy things, which increases the transactional nature rather than the social nature of the interactions among community members. Online anonymity and information asymmetry make risk-reducing signals highly influential for ties’ emergence in such a context. Specifically, in the early stage of ties emergence, due to information asymmetry and online anonymity (Mavlanova et al., 2012; Dong et al., 2022), buyers usually rely on informative signals that best express themselves to form diverse but quickly diminished portfolio of ties with sellers, which brings the “social bubbles” into the whole social commerce community (Kim, 2013). However, the development of the ties in the later stage tells an entirely different story. As buyers intend to maintain more extended ties with sellers, the transactional nature of ties diminishes, and the social nature increases. Even though most members are strangers in such an online context, at the end of the day, group norms will form and serve as the major antecedent to keep the ties longer. These research findings provide strong evidence of instabilities of buyer–seller ties in the social commerce community, suggesting to managers that the best way to keep ties with buyers in the social commerce community is to build ties with their followers or followees so that the group norm will motivate them to maintain the already existing buyer–seller ties.

### Limitations and future research

We wish to outline some limitations and points that deserve future research. First, we draw conclusions from an online social commerce community focusing only on “clothing.” Although it avoids the potential heterogeneities caused by varied product categories, it is still important to explore the boundaries of network closure mechanisms across different social commerce communities that focus on various product categories. For example, the effect of informative signals may be more salient even in the long run of ties evolution in a “digital product” community due to its functional-oriented community feature, while the effect of the social norm may be enhanced in a “tourism” community due to its social and experiential community feature. Second, many censored samples did not form ties with others during our observation period. This may be due to a relatively short observation period, especially for people who join the community later in our observation, or to the heterogeneity that certain buyers look around and intend not to form ties with anyone. Although the unobserved heterogeneity parameter in our model accounts for part of the confoundings, future research should extend the observation period to diagnose the intrinsic factors and hazard rates for these censored samples. Third, all the variables in our study are measured based on the community members’ behaviors. Although this observational study provides well-fitted models to account for the ties evolution in such an online social commerce community, we still do not know much about the mental processes the members go through in their ties strategies. By leveraging designed experiments, future research can apply a multimethod analysis to explore the actual mental processes of buyers under different ties development stages, which can provide a more comprehensive picture of the causalities between the network closure factors and ties evolutions among the social commerce community.

## Data availability statement

The original contributions presented in the study are included in the article/supplementary material, further inquiries can be directed to the corresponding author.

## Author contributions

HZ, XH, SZ, and MG conceptualized the manuscript. HZ wrote the first complete draft. XH contributed further analysis. SZ and MG contributed additional writing. All authors contributed to the article and approved the submitted version.

## Funding

This work was supported by National Natural Science Foundation of China awarded to HZ (no. 72102063) and XH (no. 72202166).

## Conflict of interest

The authors declare that the research was conducted in the absence of any commercial or financial relationships that could be construed as a potential conflict of interest.

The reviewer SS declared a shared affiliation with the author HZ at the time of review.

## Publisher’s note

All claims expressed in this article are solely those of the authors and do not necessarily represent those of their affiliated organizations, or those of the publisher, the editors and the reviewers. Any product that may be evaluated in this article, or claim that may be made by its manufacturer, is not guaranteed or endorsed by the publisher.

## Footnotes

^1^The end of a tie can usually be defined from different standards, we will illustrate our definition and the associated measurements in the data and method section.
